# Sampling of microplastics at a materials recovery facility

**DOI:** 10.1007/s00216-024-05231-x

**Published:** 2024-04-01

**Authors:** Abigail P. Lindstrom, Joseph M. Conny, Diana L. Ortiz-Montalvo

**Affiliations:** Materials Measurement Science Division, National Institute of Standards and Technology, Gaithersburg, MD USA

**Keywords:** Sampling, Environmental, Microplastics, Microscopy, Micro-Raman spectroscopy

## Abstract

Detecting, separating, and characterizing airborne microplastics from other airborne particulates is currently challenging due to the various instrumental constraints and related sample preparation hurdles that must be overcome. The ability to measure these real-world environments is needed to better assess the risks associated with microplastics. To that end, the current study focused on developing a methodology for sampling and characterizing airborne microplastics. Particulate sampling was carried out at a municipal materials recovery facility near a conveyer belt containing sorted plastic materials to collect airborne environmental particles on filters. Nucleopore filters were mounted on Teflon support rings, coated with 100 nm aluminum to reduce the background signal for micro-Raman spectroscopy, and marked with a fiducial pattern using a laser engraver. The fiducial pattern was crucial in identifying samples, relocating particles, and efficiently enabling orthogonal measurements on the same samples. Optimum sampling conditions of 2 h at 25 L/min were determined using light microscopy to evaluate the particle loadings. The filters were then cut into slices which were attached to sections of thin beryllium-copper sheeting for easy transfer of the filter between microscopy platforms. Scanning electron microscopy was used to identify carbon-rich particles. Light microscopy was used to identify colored particles which were also carbon-rich which were then analyzed using micro-Raman spectroscopy to identify specific polymers.

**Supplementary Information:**

The online version contains supplementary material available at 10.1007/s00216-024-05231-x.

## Introduction

Environmental plastic pollution is a growing problem. It’s been estimated that there have been 8300 million metric tons of plastic produced as of 2017 and production is expected to reach 12 billion metric tons by 2050 [1]. Plastics start out as primary plastics made up of large objects such as plastic bottles or bags and range in size down to nanometer-sized particles in personal care products. They undergo photochemical and mechanical weathering [2, 3] to produce smaller particles or secondary plastics. Microplastics (MPs) [3] are defined as being smaller than 5 mm whereas nanoplastics (NPs) are generally considered to range from 1–100 nm [4]. Plastic particles can be found in almost every environment [5], from soils [6], oceans [7, 8], ocean sediments [9], freshwater systems [10], the atmosphere [11], to the Arctic sea ice [12] and Antarctic waters [13]. The persistent presence of plastic particles in the global food supply [14] and the environment affects marine organisms [15], livestock animals [16], and humans [17, 18]. Their potential toxic effects on human and animal health remain a subject of intense investigation and research is in its early state [19, 20].

Separating micro- and nanoplastic particles (MNPs) from other constituents in complex environmental samples has proven to be difficult due to limitations in the sampling and characterization of such low masses and small sizes [21]. While there are a variety of methods to analyze MNP particles [22], these generally involve extraction of the particles from water or soil onto a suitable substrate for analysis. Different types of filter materials can be used to extract MNPS from samples prior to analysis, including nylon, nitrocellulose, glass fiber, polycarbonate, and stainless steel, all with distinct advantages and disadvantages [23]. More recently, silicon membrane filters were developed and used as suitable substrates to filter and extract MNPs while showing comparability within different analytical methods [24]. This study aims to bypass extraction methods by collecting and analyzing MNPs on the same substrate. The decision to use polycarbonate (PC) filters as the substrate for this work was based on past experience with these filters. PC filters are commonly used in air monitoring and microscopy techniques due to their smooth and transparent surface, precise pore diameters, and durability. However, a major problem with the direct employment of PC filters is their high fluorescence background [25]. The background can be reduced with a layer of aluminum (Al) deposited on the surface [26].

The development of metrology for airborne MNPs relies on effective sampling and sample preparation.

This work presents MNP sampling and sample preparation methods that include coating commercially available polycarbonate filters with a layer of Al and engraving fiducial patterns on the coated filter. This novel approach achieves (1) reduced Raman background issues because of the Al coating [26], as well as (2) improved conductivity for scanning electron microscopy (SEM), and (3) the fiducial markers permit characterization of the same sample across multiple microscopy techniques. Carbon-rich particles were identified using SEM with energy dispersive X-ray analysis (SEM–EDX), identified as colored using light microscopy, and analyzed by micro-Raman spectroscopy to identify the polymer type. A common approach for chemical identification was tested to examine the applicability of airborne samples collected in a real-world indoor environment, specifically at a local materials recovery facility (MRF). This location was chosen since it is an indoor environment where recycled materials, including plastics, are mechanically sorted and handled via abrupt movements on conveyer belts and rotary drums. Due to the location, we expect a higher abundance of MNPs than in a more diluted outdoor environment; however, any discussion about particle concentration at the MRF is beyond the scope of this work. The scope of this work encompasses method development for sampling and sample preparation of airborne MNPs and not ambient particle concentration monitoring of the MRF indoor air.

## Materials and methods

### Filter preparation

**Fig. 1:**
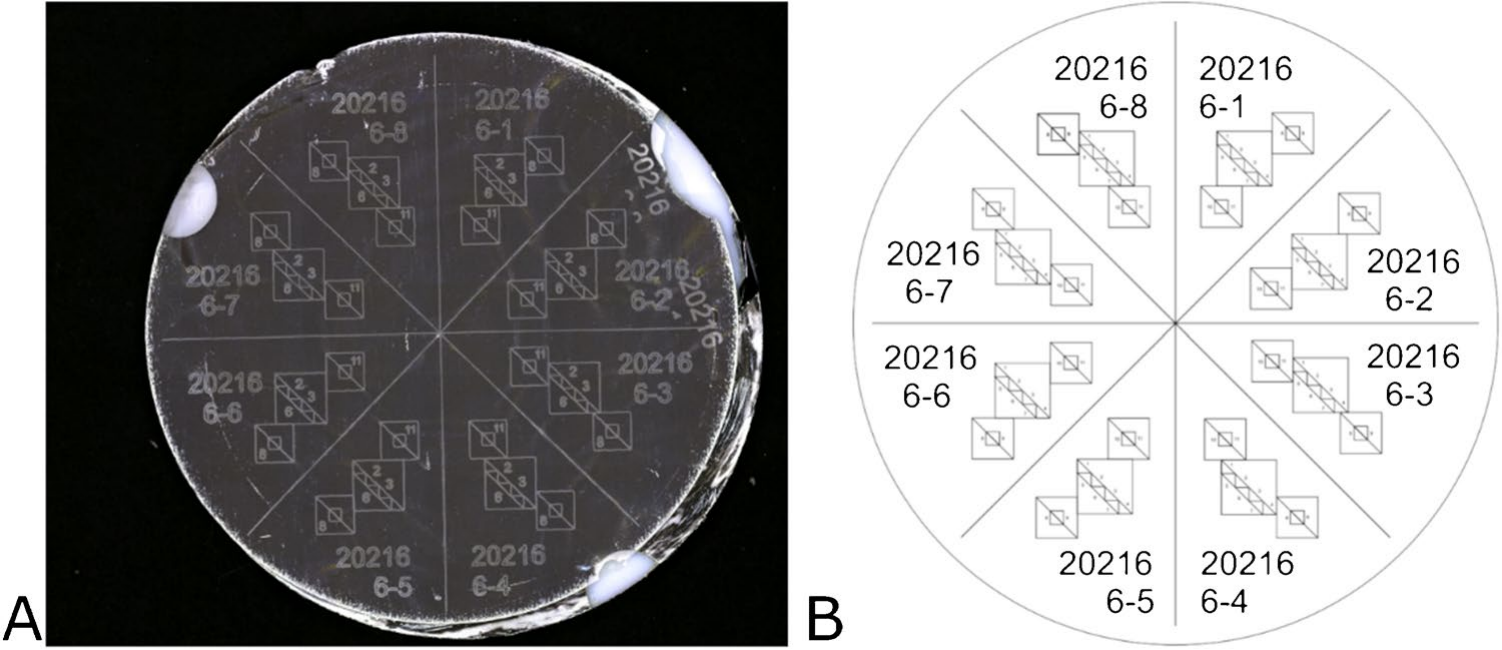
Light microscopy image of filter with fiducials (**A**) after coating and an image of fiducials showing triangles used in particle location (**B**). The light areas around the edges of the filter in (**A**) are where tabs were placed to hold the filter in the coater and the Teflon ring shows through

To prepare substrates ideal for both MP sampling and Raman characterization, Whatman (Marlborough, MA) 47-mm polycarbonate (Nucleopore) filters, with 0.4 µm diameter pores, were glued to a Teflon support ring using a thin layer of toluene-diluted contact cement (DAP Products, Baltimore, MD). (See Supplemental information (SI) for more details.) Subsequently, the filters were coated with 100 nm of aluminum using an electron beam evaporator (Denton Vacuum Infinity 22, Denton Vacuum LLC, Moorestown, NJ) to reduce the high Raman background signal from the polycarbonate filter [26]. All handling of the filters was performed with stainless steel tweezers. Finally, a fiducial pattern and sample identifiers were created using a laser engraver (Epilog, Golden, CO) to locate individual particles on filters, catalog the samples, and enable sample transfer between different spectroscopy and microscopy-based techniques (see SI for more details). Additionally, the engraving prepared for the eventual segmenting of the filter into 8 slices which will be discussed further in the next section. The total process to create a set of 15 filters took less than 6 h. Figure 1 shows an example of the completed filter prior to sampling, which includes the date that the filter was engraved as well as other identifiers and markers, as well as the design of the fiducial markers. The filters were stored until ready for use in separate 100 mm polystyrene Petri dishes. Controls were run using the same analytical equipment described in the following sections with results suggesting that no cross contamination occurred. Just before the filters were taken to the MRF for sampling, they were weighed. See SI for more details on the weighing procedure.

To have “control” particles with known composition for micro-Raman spectroscopy, pure granular or pelleted polyethylene terephthalate (PET), polyethylene (PE), high-density polypropylene (HDPE), and polystyrene (PS) were cryomilled to micrometer and submicrometer sizes. They were dispersed (see SI for more details) on an aluminum-coated filter mounted on a glass slide for analysis.

### Sampling protocol

Air sampling was conducted at MRF on a mezzanine level above a conveyer belt and rotary drum where recyclable glass, metals, and plastics first enter the MRF stream and where trash is manually removed.

For the sampling, a continuous-duty HI-Q Environmental model PSU-2 air sampler (HI-Q Environmental, Inc., San Diego, CA) with a maximum flow of 50 LPM was used. Before sampling started, the sampler’s flow rotometer was calibrated at 15 L/min and 45 L/min using a Magnehelic differential pressure gauge. The sampler’s inlet consisted of an open-faced filter holder for the Al-coated, polycarbonate membrane filter. Several inches above the inlet was a cover plate to keep excessively large particles (e.g., > 1 mm in size) from landing on the filter. The inlet extended above the railing of the mezzanine, providing a direct line of sight between the sampling inlet and the conveyer belt, about 3 m (10 feet) from the sampler. Since there was little to no size segregation, except for that provided by the cover plate, particles were collected over a large size range.

After the filter was mounted, the air sampler was turned on manually and the flow rate was adjusted. Typical air sampling conditions included one or 2 h at around 30 LPM, reduced from an initial sampling of 6 h, which produced filters that were too loaded for individual particle analysis. Table S2 in the SI shows the sampling rates and durations. For shorter sampling intervals, the sampler was timed to operate when the conveyor belt was running. Prior to removing the filter, the sampler was turned on briefly, to ensure that the flow rate had not changed. The flow rate was evaluated using the gauge on the sampler. There was no noticeable difference in flow rates with or without a filter before and after sampling, so it was concluded that the filter loading had no effect on the flow rate.

### Preparation for analysis

To calculate particle mass loading, samples were returned to the lab and weighed again using the same balance that was used to weigh the samples prior to collection. The filters were too large to fit into the field of view of the imaging instruments; therefore, they were cut into “pie” slices by rocking a scalpel blade along the etched lines between the slices (Fig. 1). Slices from the loaded filters were attached with small dabs of silver paint to a 10 mm × 10 mm section of a 0.15 mm thick beryllium copper (BeCu) sheet. The thin 10 mm × 10 mm sections allowed the sample slices on the BeCu sheet to be easily mounted on a glass slide for light microscopy and micro-Raman spectroscopy, and on aluminum stubs for SEM.

Prior to light microscopy and SEM, some filter slices were coated with ≥ 20 nm of carbon from a Cressington Carbon Coater (Cressington Scientific Instruments, Watford, UK) to minimize the charging of particles by the electron beam in SEM. Carbon coating also helped to provide a visual contrast of the particles in light microscopy.

### Imaging

**Fig. 2:**
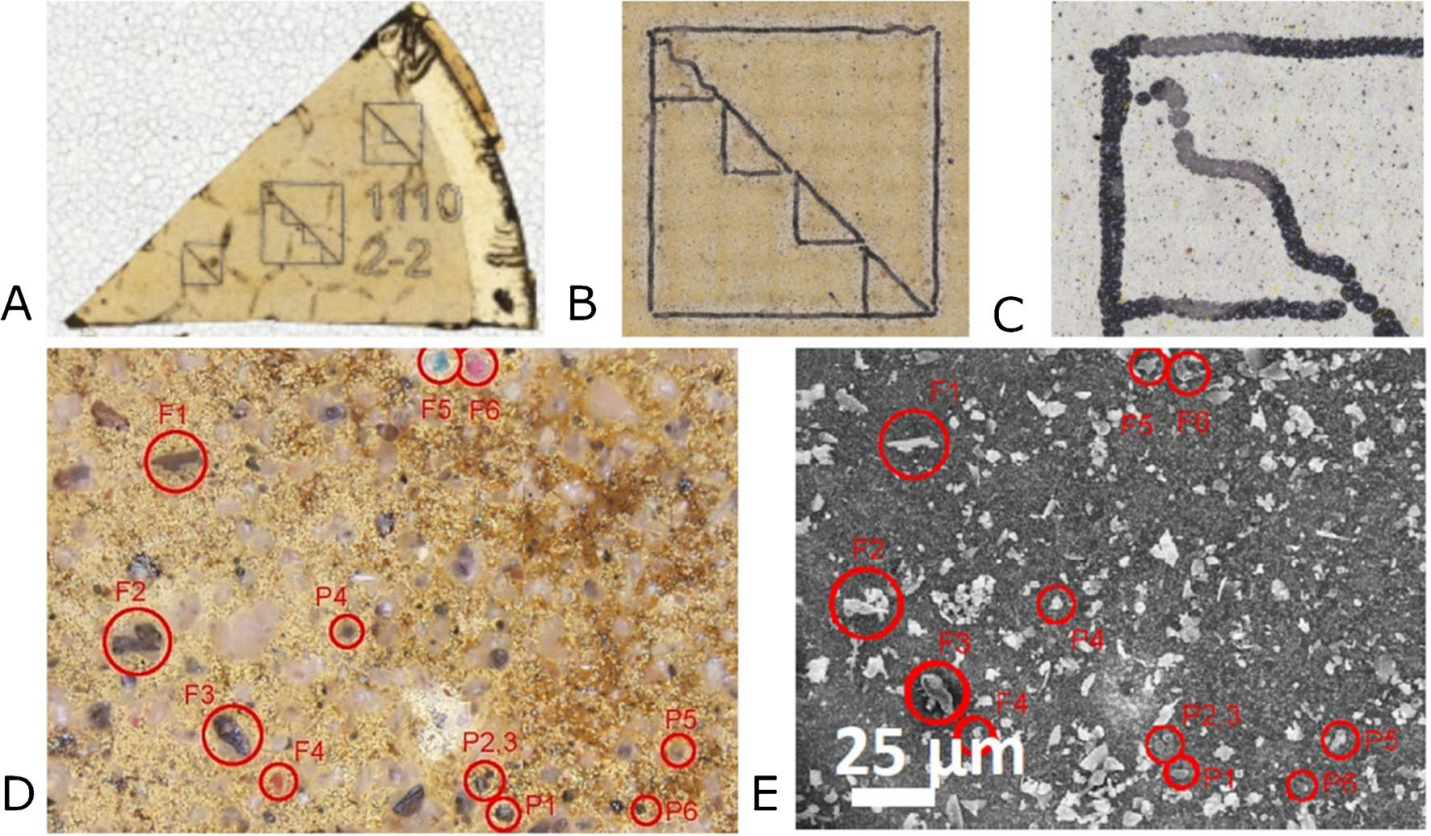
Light microscopy images of a slice of the filter (**A**), a square fiducial pattern with smaller triangles (**B**), a triangle (**C**) corresponding to the upper left triangle seen in panel B, and an image of the same location on a filter using light microscopy (**D**) and SEM SE imaging (**E**) showing the particles of interest (P) and particles having unique features (F) that were also used in particle relocations. Note that the irregular line shown in **B** and **C** aids in identifying the relevant triangle

Light microscopy was used to qualitatively inspect the population of particles by color, size, and shape in addition to visual inspection of filter loading to determine optimal sampling conditions (i.e., flow rate and sampling times). Images were acquired using a semi-motorized Olympus BX53 upright microscope in bright-field reflected mode. Image stitching and focus stacking were performed with Olympus cellSens Dimension 1.17 software. Lower magnification (× 10 objective) stitched images were taken of the filter slices and the large squares shown in Fig. 1A and B. Higher magnification (× 50 objective) stitched images with extended focal imaging were taken of each individual triangle inside the large square (Fig. 1C). Qualitative assessments using the light microscope were focused on the areas of the individual triangles in the fiducial pattern. Figure 2 shows how the image can be zoomed in to show an individual triangle and the particles in the triangle.

A coated filter that had not been taken to the MRF but was handled in the same manner as the sampled filters was imaged using the light microscope to check for PS particles on the filter.

SEM–EDX was conducted to qualitatively identify carbonaceous particles as potential MPs within a fiducial triangle on a filter slice. The carbon coating adds an insignificant signal to the total carbon EDS signal according to modeling [27]. Particles were manually selected for analysis based in part on particle size and morphology using secondary electron (SE) imaging. EDX spectra were collected with an Oxford X-Max detector (Oxford Instrument USA, Concord, MA) on a Nova NanoLab 600 FEI-focused ion-beam SEM instrument (Thermo-Fisher Scientific, Waltham, MA) for 180 s with an electron beam energy of 20 kV and current of 0.62 nA.

### Micro-Raman spectroscopy

Sample particles were analyzed with a Kaiser Optical Systems RXN1 Microprobe. In addition to sample particles, control particles from cryomilling of reagent polymer resins were analyzed.

Cryomilled “control” particles of PS, PP (polypropylene), and HDPE and a bulk sample of PET were analyzed with a Kaiser Optical Systems RXN1 Microprobe (Endress + Hauser USA, Greenwood, IN, US) fitted with a 785 nm Coherent SureLock diode laser (Coherent, Inc., Santa Clara, CA, US) attached to a Leica (Wetzler, Germany) DM2700P Microscope operating at 17 mW or 70 mW (5% or 20% of maximum power at 350 mW, respectively). For each sample, the spectrum consisted of 20 accumulations of Raman signals each collected for 5 s or 30 s and corrected for dark current.

The elevated spectral baseline caused by fluorescence was corrected in Spectragryph-id-on Ver. 1.2.15 software (https://spectroscopy.ninja) with an adaptive baseline having a coarseness setting of 15 and an offset of 0. Spectra were also smoothed in Spectragryph-id-on using the Savitsky-Golay algorithm with an interval of 50 and polynomial order of 3.

## Results and discussion

A key factor in this work is that it allows for the analysis of individual particles on filters across microscopy platforms (light microscopy, SEM, and micro-Raman spectroscopy) using the fiducial pattern with associated triangles to efficiently relocate particles between different instruments without the need to transfer the particles between different substrates. The focus of the work was colored, carbon-rich particles, since they were easy to detect with light microscopy. Preliminary Raman results on colored carbon-rich airborne particles showed that it is possible to identify specific polymers in samples from an MRF.

The mass of the filter samples as well as the flow rates is shown in the SI. The particulate loading of the filters was monitored by light microscopy, and it was determined that a shorter sampling time was needed. A 6-h sampling produced a filter that was too heavily loaded for individual particle identification, whereas either a 1-h sampling at 35 LPM or a 2-h sampling at 25 LPM had similar loadings. The 2-h sampling at the lower flow rate of 15 LPM was preferable (Fig. 2). At higher flow rates, the filter surface also became irregular due to the honeycomb surface of the filter holder screen, which made automated light microscopy and SEM more difficult. Given the location of the sampler in the MRF, the filters showed much more clear particles, and fewer plastic particles than expected. Nevertheless, this study was done in a MRF where the proportion of plastics in the indoor air is expected to be higher than in outdoor air samples. Based on SEM–EDS analysis, the majority of the clear particles were determined to be glass.

**Fig. 3:**
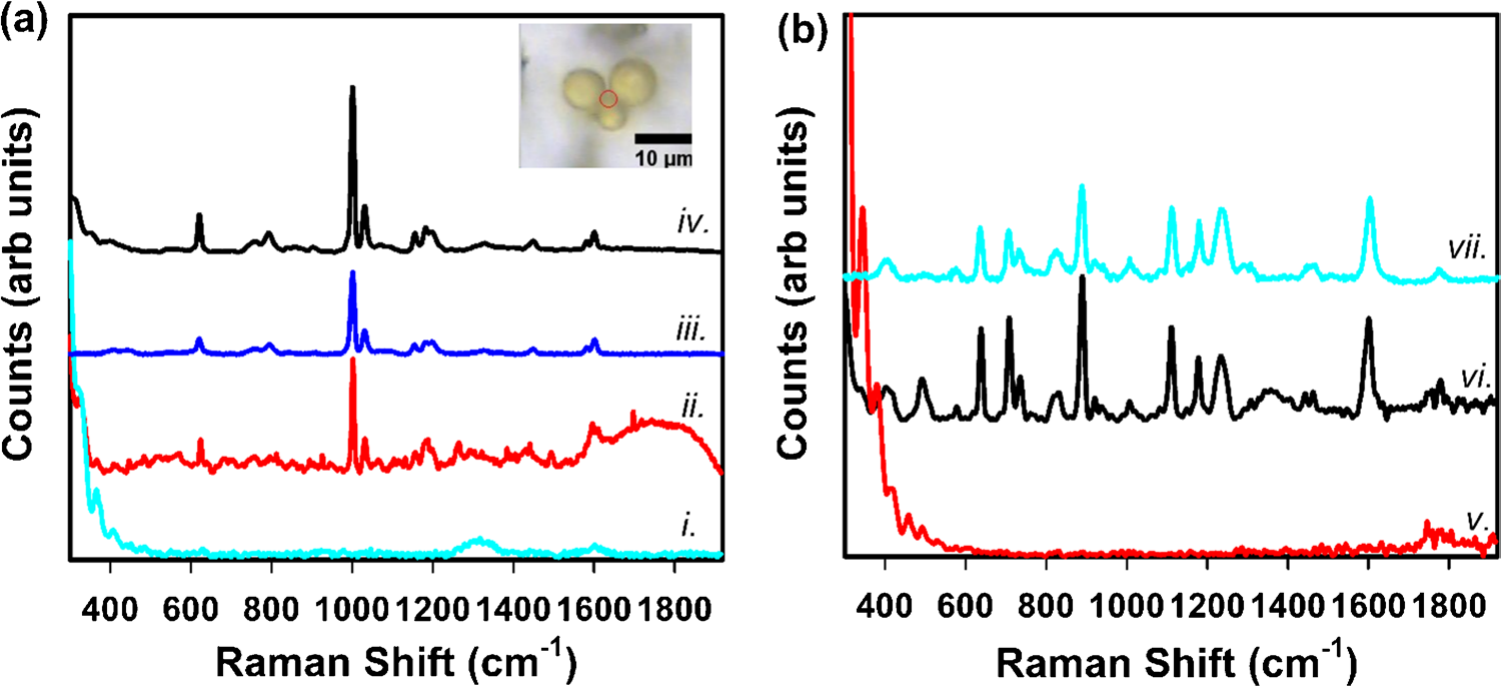
**a** Light microscopy image and micro-Raman spectra from three yellow spheres including the filter background, cryomilled PS, and a reference spectrum of PS. Note the overlap of peaks including the characteristic peak at 1001 wavenumbers. The red circle shows the location of the Raman laser spot. Also note the low background from the filter. Spectrum i shows the coated filter background, ii shows the yellow microspheres, iii shows a PS spectrum from the SLOPP library and iv shows a cryomilled PS particle reduced by 50%. **b** shows the spectra from an uncoated filter, coated filter, and polycarbonate reference spectra, all at 785 nm, to show how the coating reduces the Raman background from the polycarbonate filter. Spectrum v shows the Al-coated filter background, vi shows the uncoated filter background, and vii shows a 10 µm polycarbonate particle from the SLOPP library

Initially, carbon-rich particles were identified as candidate MPs by SEM–EDX, then, with the help of light microscopy, were relocated and analyzed by micro-Raman to confirm they were plastic particles. Figure 2 shows how the fiducials were used to identify a location on the filter starting from a slice (Fig. 2A) and moving down to a single field of view using light microscopy as well as the same field of view in SEM (Fig. 2D and E, respectively). Based on the morphology of the candidate MPs (e.g., particles of interest in Fig. 2D and E), we might expect these carbonaceous particles to result from the abrasion of bulk plastics (i.e., as secondary MPs). Additional possible sources for carbon particles could be the conveyer belt, exhaust from recycling trucks, or another source. However, none of the candidate particles inspected by micro-Raman produced spectra with identifiable polymer peaks. It is noted that the analysis was done using a 785 nm laser, and as discussed below, these particles may require a higher energy laser to increase the intensity of their polymer peak signal. Due to this challenge and the high number of carbon-containing particles identified using SEM–EDX, light microscopy was ultimately used instead to distinguish potential plastic particles based on color. The use of the fiducial pattern in the filters made it possible to go directly and quickly from light microscopy to micro-Raman and skip the time-consuming step of using SEM–EDX to identify candidate MPs. SEM–EDX was still used to complement the light microscopy and micro-Raman analysis since it provides additional information about particle populations, especially the inorganic particles. Also, SEM–EDX is useful because it is not biased towards colored and spherical particles, whereas inspection with light microscopy potentially misses white or colorless particles. Figure 3a shows micro-Raman spectroscopy and light microscopy of 3 yellow spheres as well as the background from the coated filter, a cryomilled PS particle, and a PS reference spectra [28]. The matching peak at 1001 cm^−1^ is characteristic for PS. The low background from the filter and the collection of Raman spectra from the small particles, where both the cryomilled particle and the MRF particles are less than 10 µm, show that identification of MPs in MRF samples is possible with further work. The lowering of the background from the filter is further illustrated in Fig. 3b, which shows an Al-coated filter, uncoated filter, and polycarbonate reference spectra. The source of the spherical MPs is currently unknown and is the focus of future research. While NP can be collected, characterization is difficult because the Raman signal is not strong enough for particles smaller than a few micrometers in diameter due to high signal to noise.

One of the main benefits of the sampling and filter design is minimizing sample preparation that allows direct analysis on the filter, and which minimizes processes that can affect composition or morphology changes such as when using solvents or mechanical forces. The method developed in this study has some limitations and needs further improvements, despite its obvious benefits. One of the main limitations of this method is that the emphasis on light microscopy to identify potential plastic particles based on color makes it hard to detect white and uncolored particles, especially due to the high number of clear or colorless particles on the filters. This limitation will be addressed in a future study looking at using SEM–EDX identification of carbonaceous particles in conjunction with polymer identification using a micro-Raman system with multiple wavelengths, particularly one at higher energy, which would allow potentially unidentified MPs to produce higher intensity signals. Furthermore, an automated micro-Raman system would allow for higher throughput analysis.

Another limitation is that the method cannot detect or characterize plastics in the nanometer range due to the limitations of the micro-Raman instrument. Also, the sampling procedure lacks size segregation, making it difficult to focus on a particular size range of particles. Size segregation could be provided by using a different filter sampler, such as an impactor. Another benefit of this procedure is that it provides a complex environmental sample of MPs without a tissue or water matrix that, with further analysis, could be used as a proxy for controlled environmental studies. In addition, sampling at a site such as a materials recovery facility provides a type of sample where the concentration of plastics is expected to be high and, therefore, advantageous for studying environmental plastics. Lastly, this method enables the easy and time-saving relocation of individual particles between different microscopy techniques.

### Supplementary Information

Below is the link to the electronic supplementary material.

Supplementary file1 (DOCX 2.15 MB)
