# Supplementary material for: Sampling of microplastics at a materials recovery facility
Source: Anal Bioanal Chem. 2024 Apr 1;416(12):2885–91. doi: 10.1007/s00216-024-05231-x (PMC11045654; doi:10.1007/s00216-024-05231-x)
Supplement: Supplementary file 1 — Supplementary file1 (DOCX 2.15 MB) [file 216_2024_5231_MOESM1_ESM.docx]

**Supplemental Information for Sampling of Microplastics at a Materials Recovery Facility**

*Analytical and Bioanalytical Chemistry*

Abigail P. Lindstrom (0000-0002-1730-257X), Joseph M. Conny (0000-0002-0553-3127), and Diana L. Ortiz-Montalvo (0000-0001-7293-4476)

Materials Measurement Science Division, National Institute of Standards and Technology, Gaithersburg, MD

Corresponding author: [Abigail.Lindstrom@NIST.GOV](mailto:Abigail.Lindstrom@NIST.GOV)

Filter Preparation

The filters were attached to Teflon rings to provide stability while sampling and during handling. Each 47 mm diameter Teflon ring (Hi-Q Environmental, San Diego, CA) was placed in a ring stand clamp turned so that it can be held horizontal. A cotton swab (Top Care, Elk Grove Village, IL) was used to spread the entire surface of one side of the ring with a solution of Contact Cement (Wildwood Contact Cement, DAP Products, Baltimore, MD) diluted with toluene (EMD Chemicals, Wentworth, GA). It was then let sit for 5-10 minutes to let the cement become tacky (Figure S1). While the cement was becoming tacky, a Whatman 47 mm nucleopore filter, with 0.4 µm pores, was picked up with tweezers and placed upside down (shiny side down) on one of the light blue protective rounds that came with the filter on top of a filter holder base on top of a filtration flask. The arm of the flask was attached via tubing to the house vacuum system. The vacuum was then turned on just enough to provide enough suction to hold the filter flat. After the cement was tacky, the ring was removed from the clamp and turned upside down against the back of the filter being careful to put the ring around the outside of the filter. It was left for another 5-10 minutes to make sure that the filter was completely attached to the ring. The filter was then removed and placed in an individual Petri dish. Figure S2 shows a SEM Seccondary Election (SE) image of the coated and uncoated filters showing the pores in the filter which are still visible on the coated filter.


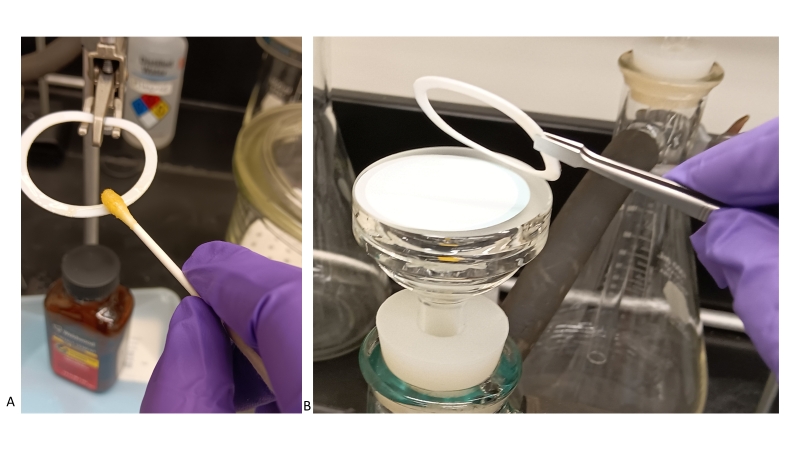


**Fig. S1** Spreading dilute contact cement on a ring. The color of the cotton swab is caused by the contact cement. (B) Placing the ring on the back of the filter. The filter is offset on the protective sheet to show both the filter and the protective sheet.


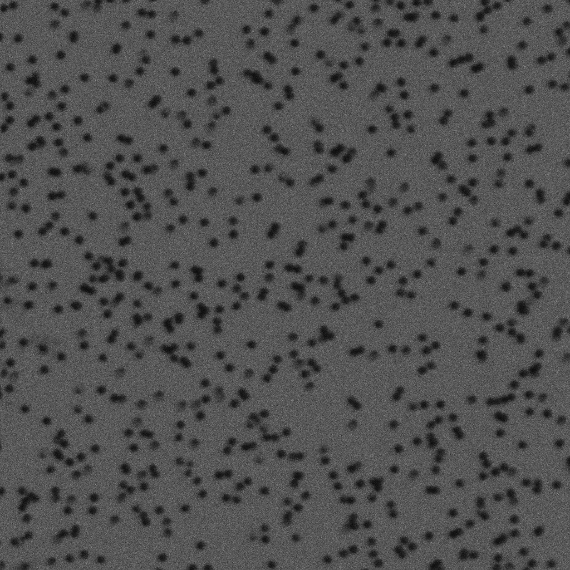

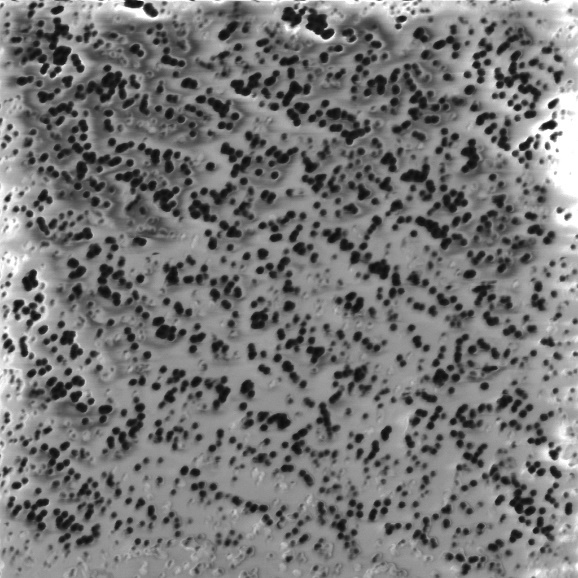


**Fig. S2** SE images of coated (left) and uncoated (right) filters. The field of view of the coated filter is 25 µm and in the uncoated filter 50 µm. The light areas in the uncoated filter are caused by charging in the SEM.

Engraving

Once coated, the filters were engraved with a fiducial pattern (Figure 1). The template for the printing was created in Corel Draw (Alludo, Ottawa, Ontario, Canada) and the engraving was done on an Epilog Laser Engraver (Epilog, Golden, CO). The filter was removed from the Petri and placed in the upper left of the engraver, pushed gently against the top and a slight distance to the right of the left edge. The laser head was lowered until the spacer was just above the filter, but not touching it, the spacer was removed and the top closed. The template for the printing was created in Corel Draw starting with a circle 1.85 inches across. It was divided into 8 slices, then the squares, triangles and labels were added. Once the filter was added and the template generated, the template was printed onto the filter. It’s important that the engraver use a short dwell time, low power, and relatively high speed to avoid damaging the filter. Table 1 shows the settings for the Epilog Laser. Once the engraving is completed, the sample is removed and placed back in the Petri. The filters were engraved individually, although it would be possible to engrave more at a time, but the set up would be more involved and the location and the spacing of the extra filters would have to be carefully monitored. After engraving, the filters are weighed. Once the template was set up and conditions finalized, a filter could be engraved in a matter of a minute including placing of the filter, engraving and removing the filter.

**Table S1** Settings for Epilog Engraver

| **Raster settings** |  |
| --- | --- |
| Speed | 50% |
| Power | 50% |
| Frequency | 50% |
| Engraver Direction | Top-Down |
| Energy Filtering | Standard |
|  |  |
| **Vector Settings** |  |
| Speed | 50% |
| Power | 20% |
| Frequency | 30% |
| Power-comp vs Speed Comp | Power Comp |
| Horizontal and vertical size of sample (in) | 1.85 |

Weighing of Filters

The balance used to weigh the filters is a Fisher Gram-atic balance (Thermo Fisher Scientific, Waltham, MA) with an uncertainty of 0.01 mg. The balance is in a basement lab, so that the temperature is kept consistently at 21° C (70° F). The humidity varies a small amount over a year, but not over several days. All the filters were equilibrated in the laboratory for at least several days before weighing. The balance has an enclosed Ra-226 source that reduces electrostatic charging, which is minimal for polycarbonate filters. It also sits on a granite weighing table which reduces environmental vibrations. Before each weighing session, the balance is zero’ d and is checked with a known weight. A single replicate was measured for each filter pre and post-sampling.

Preparation of Known Materials

The cryomilled particles were suspended in a solution of 3% surfactant (w/w) and 4% water in ethanol. A drop of the suspension was dispersed from a capillary tube onto a piece of Al-coated filter. It was dried without heating since the ethanol evaporated quickly and didn’t leave a visible residue. The surfactant used was 3,5-dimethyl-1-hexyn-3-ol.

Filter Masses

**Table S2** The flow-normalized masses are intended to account for times when the conveyer belts are shut down due to employee breaks. There seems to be little relationship between the time of day and filter loading, except for the overnight blank.

| Sample group ID | Collection date | Operating interval | Nominal collection duration (min)^1^ | Particle mass (mg) | Flow rate (LPM) | Flow-normalized particle mass (μg)^2^ | Mass above or below average | Example Images |
| --- | --- | --- | --- | --- | --- | --- | --- | --- |
| 211012-4 | 10/21/2021 | 7 am to 1 pm | 295 | 5.5 | 35 | 0.536 | above (>2 s) | 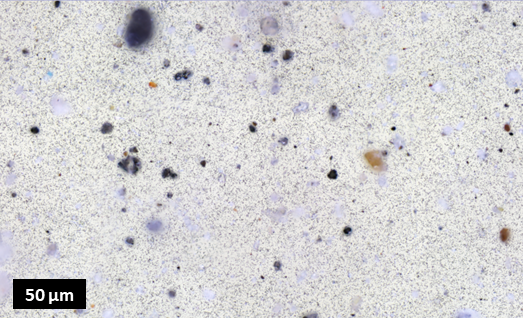  *Light loading: 211110-9*^4^  *(1 h, 35 LPM)*  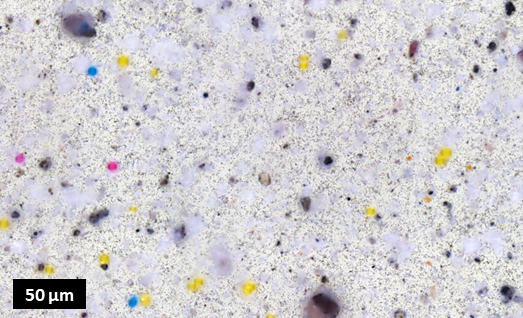  *Medium Loading: 211110-2 (2 h, 15 LPM)*  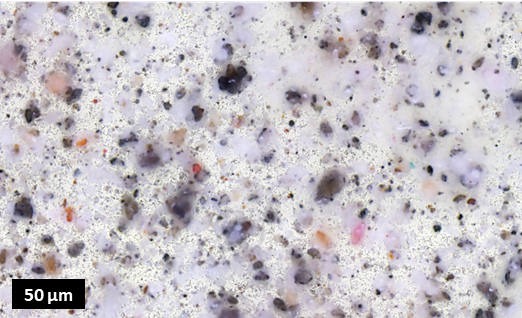  *Heavy Loading: 211110-1*  *(2 h, 45 LPM)* |
| 211012-5 | 11/4/2021 | 7 am to 1 pm | 295 | 2.2 | 25 | 0.299 | below |  |
| 211012-7 | 10/29/2021 | 7 am to 1 pm | 295 | 2.7 | 35 | 0.265 | below |  |
| 211012-9 | 11/3/2021 | 7 am to 1 pm | 295 | 2.1 | 25 | 0.280 | below |  |
| 211012-11 | 11/10/2021 | 7 am to 1 pm | 295 | 1.7 | 15 | 0.393 | above |  |
| 211012-12 | 11/18/2021 | 7 am to 1 pm | 295 | 1.4 | 15 | 0.316 | below |  |
|  |  |  |  |  |  |  |  |  |
| 211110-1 | 11/24/2021 | 9 am to 11 am | 120 | 2.1 | 45 | 0.393 | above |  |
| 211110-2 | 12/2/2021 | 9 am to 11 am | 120 | 0.6 | 15 | 0.350 | above |  |
| 211110-3 | 12/8/2021 | 9 am to 11 am | 120 | 1.5 | 30 | 0.417 | above |  |
| 211110-4 (facility idle) | 12/8/2021  12/9/2021 | 11 pm to 5 am | 360 | 0.4 | 25 | 0.042^3^ | below (>2 s) |  |
| 211110-5 | 12/15/2021 | 9 am to 11 am | 120 | 1.1 | 25 | 0.370 | above |  |
| 211110-6 | 12/22/2021 | 12 pm to 1 pm  1:30 pm to 2:30 pm | 120 | 0.8 | 30 | 0.211 | below (>1 s) |  |
|  |  |  |  |  |  |  |  |  |
| 220216-3 | 4/26/2022 | 9 am to 11 am | 120 | 0.6 | 20 | 0.255 | below |  |
| 20216-4 | 4/27/2022 | 9 am to 11 am | 120 | 0.5 | 35 | 0.126 | below (>1 s) |  |
|  |  |  |  |  |  |  |  |  |
|  |  |  |  |  | Average^3^ | 0.324 |  |  |
|  |  |  |  |  | Standard  Deviation^3^ | 0.104 |  |  |

^1^ Collection duration omits time when facility was idle due to staff breaks.

^2^ Particle mass normalized for flow rate and collection duration (mass per L air volume).

^3^ Average and standard deviation does not include sample 211110-4 when facility was idle.

^4^ Sample 211110-9 was not weighed but the image is shown as a representative lightly loaded sample.
